# Enhanced novelty detection in auditory scenes through adaptation of inhibition

**DOI:** 10.1186/1471-2202-16-S1-P72

**Published:** 2015-12-18

**Authors:** Bertrand Fontaine

**Affiliations:** 1 Laboratory of Auditory Neurophysiology, Faculty of Medicine, University of Leuven, Leuven, Belgium

## 

Animals and humans must behave efficiently in acoustically rich environments, where few sound sources are of interest out of many others that are considered background noise. How does the auditory system implement such robust processing is a topic of intense research. In this work I am interested in the neural mechanisms underlying detection of novel objects in auditory scenes, such as a novel sound source at a given location.

It has been shown psycho-physically that the perception of a sound depends on the acoustical context in which it occurs. A basic example of such effect is called enhancement [1]. A harmonic complex (i.e. a series of tones at different frequencies) presented in isolation will be perceived as a single auditory object. If one tone is deleted from the complex (the conditioner) and then reintroduced (test), it will perceptually "pop out" as a separate sound object. To maximally induce enhancement in humans, the conditioner must have an energy notch in its spectrum with a preferred width of 0.6 octave. This effect originates in the central nervous system; neural correlate thereof were found in the cochlear nucleus, two synapses away from the cochlea [2]. The standard model explaining the emergence of auditory enhancement is based on the adaptation of wide-band inhibition inputs [1,2]: the increase in output rate due to a preceding conditioner comes from a decrease in activity of the wide-band inhibitory inputs, i.e. inhibition have adapted during the conditioner while narrow band excitation has not. In this study I seek to test functions of enhancement effects in realistic listening situations. In particular, I hypothesize that the enhancement of speech signals by realistic environments is a neural implementation of a novelty detector.

I implemented on the Brian simulator and its auditory package [3] a model of the auditory periphery followed by a network of adapting spiking neurons [4] comprising feed forward excitation and inhibition. This network was first tuned to reproduce the basic enhancement effects reported physiologically. In order to mimic realistic listening conditions, sounds are filtered by realistic environmental processes prior to being fed to the model. In particular I'm interested in the filtering induced in sounds arriving at the ear by the listener's body and by the surrounding room. The head and the shoulder create reflections of the direct sound, resulting in notches in the high frequency sound spectrum, which depend on the sound source elevation [5]. Similarly, reflections from the ground and the walls of a room will induce notches in the low-frequency spectrum, which depend on the sound source azimuth and distance [6]. As spectral notches are critical in inducing auditory enhancement I show that if a novel sound is preceded by other sounds located at different positions, the detection of this novel sound will be enhanced.

The present functional study of auditory enhancement suggests that such contextual effects could be critical for animal survival and for hearing in realistic conditions. The results show how simple neural mechanisms modulate a sensory pathway so that it adapts to its surrounding environment and suggest that bias in perception could have ecological advantages. The model could also inspire the design of speech processing devices in real environments.
